# Improvement of Biophysical Skin Parameters of Topically Applied Fermented Soybean Extract-Loaded Niosomes with No Systemic Toxicity in Ovariectomized Rats

**DOI:** 10.3390/pharmaceutics13071068

**Published:** 2021-07-12

**Authors:** Wandee Rungseevijitprapa, Bancha Yingngam, Chaiyavat Chaiyasut

**Affiliations:** 1 Department of Pharmaceutical Chemistry and Technology, Faculty of Pharmaceutical Sciences, Ubon Ratchathani University, Ubon Ratchathani 34190, Thailand; bancha.y@ubu.ac.th; 2 Innovation Center for Holistic Health, Nutraceuticals and Cosmeceuticals, Faculty of Pharmacy, Chiang Mai University, Chiang Mai 50200, Thailand

**Keywords:** fermented soybean extract, niosomes, ovariectomy, skin aging, toxicity

## Abstract

Despite the known beneficial impacts of estrogen used as hormone replacement therapy to ameliorate signs of skin aging in postmenopausal women, its compliance rates are low. A significant amount of estrogen may be absorbed into the blood circulation and can lead to systemic actions. Soy isoflavone exhibits biological activities similar to synthetic estrogen because it is a heterocyclic phenolic compound. The disadvantage of most topical ingredients based on isoflavone is that they contain biologically inactive glycoside forms, which must be converted to a readily absorbed aglycone for the topical application. The purposes of this study were to develop niosomes-loaded *Aspergillus oryzae*-fermented soybean extract (FSE) to enhance skin absorption with proven systemic side effect compared to estrogen application. Skin hydration and viscoelasticity of 75 days post-ovariectomized (OVX) Wistar rats following 84-day topical treatment with various tested gel formulations containing fermented soybean extract (FSE) were evaluated. The tested formulations were gel + FSE nanoniosomes, gel + FSE microniosomes, gel + FSE (200 µg FSE/9 cm^2^/rat), gel + blank nanoniosomes (a negative control), and gel + 17β-estradiol (E2) nanoniosomes (a positive control, 20 µg E2/9 cm^2^/rat). Changes in vaginal cornifications and weights of uteri, livers, and kidneys in the OVX rats and signs of primary skin irritation in the rabbits were evaluated for their toxicities. Results showed that FSE-loaded nanoniosomes improved the skin hydration and viscoelasticity better than gel + FSE microniosomes and gel + FSE, respectively, but lower than those of gel + E2 nanoniosomes (*p* < 0.05). Unlike all gel + E2 nanoniosomes, the FSE formulations showed no changes in vaginal cells and weights of uteri, livers, and kidneys and no signs of skin irritation. In conclusion, The FSE niosome-based gels should be promising candidates for delivering phytoestrogens against signs of skin aging with no systemic toxicities.

## 1. Introduction

Since estrogen plays a significant role in the modulations of skin physiology, insufficient estrogenic levels during post-menopausal physiological changes result in the reduction of dermal and epidermal cellularity and collagen quantity [1]. Consequently, skin aging, which appears as vaginal atrophy, dryness, and wrinkles, becomes noticeable due to changes in the structural architecture of the skin, including decreased collagen content, decreased elasticity, increased wrinkling, and increased dryness [2]. Hormone Replacement Therapy (HRT) of 17β-estradiol applied orally or topically can ameliorate signs of skin aging by increasing skin elasticity and hydration, reducing wrinkles and collagen synthesis [3,4,5]. Despite the positive effects of estrogen as HRT in managing menopausal symptoms and reversing skin physiology, there remains controversy over its long-term health risks and connection to breast cancer [6].

In recent years, phytoestrogens have gained considerable attention as an alternative to estrogen because of their few or no adverse effects due to their rapid clearances from plasma [7]. Among several types of phytoestrogens, soy isoflavones and their health benefits have been published in over 10,000 peer-reviewed scientific articles. Despite the well-documented use of soy isoflavone as a dietary health supplement administered orally, long-term consumption of isoflavones may associate with potential carcinogenic and immunosuppressive effects raise concerns. Topically applied isoflavone is an interesting pathway to bypass such systemic risk; there is still a limited use for skin application [8,9]. Most soybean extracts generally contain isoflavone glycoside conjugates as the predominant non-active forms [10]. Bioconverting isoflavone glycosides into their corresponding and readily absorbed aglycones can be facilitated by using solid-state fermentation of soybeans with *Aspergillus spp.* During the fermentation process, the fungi secrete the β-glucosidase enzyme to hydrolyze β-glycosidic linkage between aglycone and sugar moiety of isoflavone glycosides resulting in higher content of isoflavone aglycones [11].

Fermented soybean extract (FSE) has been reported to have better in vitro activities for phytoestrogenic, antioxidative, and anti-non-enzymatic protein glycations than the non-FSE [12,13,14]. However, dermal delivery of isoflavone aglycone applied in conventional skincare dosage forms into viable epidermis and dermis through the stratum corneum is still limited because of low skin absorption through the stratum corneum [15]. This detrimental effect leads to limited use of soybean extract in skincare products. To solve the mentioned drawbacks, niosomes are a promising strategy to enhance the skin penetration of phytoestrogenic glycoside. Niosomes are lamellar (bilayer) structures based on non-ionic surfactants and cholesterol, which offer higher chemical and physical stability [16].

Niosomes have also shown many other potential benefits, including protecting active compounds from degradation, maintaining a sustained release, improving penetration of active compounds into the skin, and reducing skin irritation [17]. Topically applied niosomes can enhance the residence time of active compounds in the stratum corneum and epidermal skin [16,17,18,19,20,21]. Although soy isoflavone has been proven to be safe as a nutritional product [22,23], there was no report on the potential benefit and risk of systemic side effects of topically applied FSE-loaded niosomes compared to synthetic estrogen therapy.

The objectives of this study were to develop niosomes containing phytoestrogenic glycoside and its corresponding aglycone from soybean extract for topical skin delivery. Niosomes in micro-and nano vesicle size range containing various FSE-based gels against signs of skin ageing were investigated. Potential systemic toxicities of the FSE niosome through dermal absorption were also evaluated in comparison to those of the synthetic estrogen formulation in ovariectomized (OVX) rat model. Primary skin irritations were assessed in rabbits.

## 2. Materials and Methods

### 2.1. Materials

The following chemicals were obtained from commercial suppliers and used as received: sorbitan monostearate (Span60), E2, genistein, (Sigma-Aldrich, St. Louis, MO, USA), cholesterol, sodium chloride, sodium lauryl sulfate (Carlo Erba Reagents SpA, Cornaredo, MI, Italy), Zoletil100 (tiletamine and zolazepam) (Virbac Laboratories, Carros Cedex, France), *Aspergillus oryzae TISTR 3018* (Thailand Institute of Scientific and Technology Research, Khlong Luang, Pathum Thani, Thailand), and soybean seeds cultivar Sorjo-II (*Glycine max* (L.) Merrill) (Chiang Mai, Thailand), Dulbecco’s Modified Eagle’s Medium (DMEM), phenol red-free DMEM, fetal bovine serum (FBS), dextran-coated charcoal FBS (Gibco Invitrogen Corporation, Carlsbad, CA, USA). Absolute ethanol was obtained from Carlo Erba Reagent. All chemicals were of analytical grade.

### 2.2. Preparation of the FSE

The FSE was prepared using a solid-state fermentation according to the method described previously [21] with slight modifications. Briefly, the spore suspensions of *A. oryzae* (1 × 10^6^ spores/mL) were sprayed onto the surface of the sterile soybean flour at a ratio of 1 mL per 50 g of soybean and incubated at 30 degrees Celsius (°C) for 10 days. The fermented soybean was defatted with hexane and extracted in ethanol using a Soxhlet apparatus. The obtained ethanolic mixture was cooled and filtered through a 0.2-micrometer filter membrane to remove insoluble substances. The ethanol was then removed under reduced pressure at 45 °C, and the extract was then dried using a freeze dryer (Martin Christ GmbH, Osterode am Harz, Germany). The resulting dried extract with a percent yield of 13.47% *w*/*w* was kept at −20 °C until used.

### 2.3. Quantification of Isoflavone Amounts

The isoflavone amounts in FSE were determined using high-performance liquid chromatography (HPLC) (Waters Corporation, Milford, MA, USA) modified from Chuankhayan et al. [24], at 255 nm, equipped with a symmetry C18 column (3.9 × 150 mm, 5 μm) (Waters Corporation, Dublin, Ireland). The mobile phase comprised of 0.1% (*v*/*v*) phosphoric acid in water (A) and acetonitrile (B) with a flow rate of 1 mL/min. The following gradient program was used: 90%A–10%B (3 min), 65%A–35%B (40 min), 50%A–50%B (2 min), 50%A–50%B (5 min), 90%A–10%B (5 min). All samples were analyzed at 25 °C. Daidzein and genistein were used as the standard markers. The calibration curve of isoflavone aglycones in FSE was also inserted in the HPLC chromatogram (see Section 3.1).

### 2.4. Evaluation of Estrogen-Like Activities

The estrogen receptor-positive human mammary adenocarcinoma (MCF-7) cells were seeded into a 96-well plate at a density of 1 × 10^3^ cells/well in DMEM containing 10% heat-inactivated FBS. The cells were allowed to attach for 48 h, and the culture medium was then removed and washed with phosphate buffer saline solution. The medium was changed to phenol red-free DMEM supplemented with 7% dextran-coated charcoal FBS. After 48 h incubation, the cells were treated with various concentrations of FSE, daidzein, genistein, or E2. Cell proliferation was measured on the sixth day that cell growth of 0.1 nM E2 reached 90% confluence using 3-(4,5-dimethylthiazol-2-yl)-2,5-diphenyl-tetrazolium bromide (MTT) assay as previously described [25,26]. The absorbance was measured at 570 nm with background subtractions at 630 nm using a microplate reader (Dynex/MRX microplate reader, Dynex Technologies, Inc., Chantilly, CA, USA). The solution of 0.1 nM E2 and 0.1% dimethyl sulfoxide served as a positive and negative control, respectively.

### 2.5. Preparation of the Test Micro-and Nanoniosomes

Blank micro-and nanoniosomes were prepared using the reverse-phase evaporation method [27], followed by particle size reduction by homogenization. Briefly, an accurately weighed amount of Span^®^ 60 and cholesterol (1:1 mM ratio) was dissolved in 20 mL absolute ethanol in a round bottom flask. Then, the organic solvent was evaporated under vacuum at 50 °C using a rotary evaporator (Buchi RotavaporVR R-100, Flawil, Switzerland), and the thin, dry film was formed in a round bottom flask. The thin-film was hydrated with deionized water and then sonicated in an ultrasonic bath (BransonVR Ultrasonic, B2210E-DTH, Danbury, CT, USA) at the ultrasonic power of 234 watts with a 50 Hz frequency for 30 min at 60 °C. Particles size in the niosomal suspension was further reduced using a high-pressure homogenizer (Invensys APV1000, Albertslund, Denmark) at 500 bars for 10–15 min to obtain the desired particle sizes. For the preparation of FSE- or E2-loaded niosomes, 1% *w*/*v* FSE or 0.01% *w*/*v* E2 (final concentration) were mixed with Span60 and cholesterol in the round bottom flask before the preparation thin film of niosomes, and the procedure was performed as the same method described above.

### 2.6. Characterization of Niosomes

#### 2.6.1. Measurement of Particle Sizes and Zetapoential and Morphology

Particle sizes and zeta-potential of niosomes were measured using a photon correlation spectroscopy (PCS) (Malvern Zetasizer Nano ZS, Malvern Instruments, Malvern, UK) as previously described by Akbari et al [28]. The samples were diluted 40-fold in deionized water before measurement. For the zeta potential analysis, the samples were measured by a laser Doppler electrophoretic mobility (Malvern Zetasizer Nano ZS, Malvern, UK). All measurements were performed in triplicate at 25 °C. Additionally, the morphology of the resulting niosomes was examined by scanning electron microscopy (S-3400 N, Hitachi, Tokyo, Japan) and atomic force microscopy (SPA-400, Seiko Instruments, Chiba, Japan). Zeta potential values of niosome suspensions were measured by laser Doppler electrophoretic mobility measurement (Malvern Zetasizer Nano ZS, Malvern Instruments, Malvern, UK). The samples were diluted with ultrapure water, and zeta potential measurements were performed from 30 runs.

#### 2.6.2. Determination of Entrapment Efficiency

The entrapment efficiency of phytoestrogenic markers (daidzein and genistein) in micro-and nanoniosomes was determined by the HPLC method as described above. Free phytoestrogens were separated by ultracentrifuge (Beckman OptimaTM Series L-90K, Fullerton, CA, USA) at 200,000× *g* at 4 °C for 1 h. According to the following equation, the entrapment efficiency (%*EE*) of each formulation was calculated by Equation (1) [20,29].

(1) $$\%\;EE=\frac{\left(a-b\right)}{a}\;\times \;100$$

where a is the amount of total phytoestrogen added to the formulation, *b* is the amount of phytoestrogen in supernatant.

### 2.7. Preparation of Test Gels

Gels were prepared using 0.2% acrylates/C10–30 alkyl acrylate cross-polymer as a gelling agent. Gels containing active ingredients, 12% deionized water were replaced with the concentrated niosome suspensions (concentrated × 10). These formulations were (i) blank nanoniosomes (288 ± 16 nm), (ii) 1% FSE nanoniosomes (285 ± 13 nm), (iii) 1% FSE microniosomes (1035 ± 43 nm) (final concentration of FSE in each gel was 1%), (iv) 0.01% E2 nanoniosomes (283 ± 8 nm) (final concentration of E2 in gel was 0.01%), and (v) 1% FSE gel. The gels were prepared under constant stirring with a propeller at 300 rpm for 10–20 min to obtain the homogeneous formulations, using a high-pressure homogenizer (Invensys APV1000, Albertslund, Denmark) at 500 bars to obtain the desired particle sizes.

### 2.8. In Vivo Animal Studies

#### 2.8.1. Animals

The experimental protocol of the present study was approved by the Ethics Committee for Use and Care of Animal of the Ubon Ratchathani University (protocol no. 18). Female Wistar rats with normal estrus cycles were studied to evaluate the in vivo anti-skin-aging, and toxicity of FSE as previously described [25]. The rats were obtained from the National Laboratory Animal Center, Mahidol University, Nakhonpathom, Thailand. All rats were separately housed in individual metabolic cages under standard experimental conditions of 24 ± 1 °C and 12 h light/dark cycle. The rats aged 10.5 months were anesthetized by intramuscular injection of Zoletil 100® (tiletamine/zolazepam) before ovary removal and ovariectomy-induced skin aging for 75 days according to our preliminary study. A vaginal smear was taken and placed on a glass slide, and a drop of normal saline solution was added. Cellular differentiation was observed under light microscopy (Nikon Eclipse E200, Nikon Instruments Inc., Melville, NY, USA), and the diestrus phase indicated a successful ovariectomy. The hair on the dorsal skin of the rats was carefully shaved with an electric shaver. The rats were randomly allocated to Groups 1–5 with 4–5 animals per group as follows: Group 1 received only gel containing blank nanoniosomes (negative control, *n* = 4); Group 2 received gel containing 1% (*w*/*v*) FSE (*n* = 5); Group 3 received gel containing 1% (*w*/*v*) FSE/microniosomes (*n* = 5); Group 4 received gel containing FSE/nanonisomes (*n* = 5); Group 5 received E2/nanoniosomes (positive control, *n* = 4). The baselines for skin parameters of each rat were monitored using a non-invasive bioengineering method for 75 days (pre-treatment period). The rats were then dermally applied with test formulations once a day at 0.2 mL/9 cm^2^ for 84 days, and changes in their skin properties were examined 15 h after application on days 7, 14, 42, 56, and 84.

#### 2.8.2. In Vivo Efficacy Studies

The hair on the dorsal area of the rats was clipped 1 week before the measurement of skin properties to minimize any potential effects of hair clippings (Electric hair clipper, T.H.K. Clipper Style, Los Angeles, CA, USA) modified from previously research [30]. Each rat was allowed to rest at least 30 min before measurement in a controlled room of 23.2 °C, 50.5% relative humidity. The skin biophysical properties were measured at baseline and after applying the test formulations by a non-invasive bioengineering method with multi-probe skin testers connected to CK-MPA and MPA 580 software (Courage & Khazaka Electronics GmbH, Cologne, Germany). The measurement was taken on days 75, 30, and 0 before treatment and on days 7, 14, 42, 56, and 84 after treatment.

##### Measurement of Skin Hydration

Skin hydration in the superficial layers at 10–20 μm thickness of the stratum corneum was determined using a Corneometer connected to CK-MPA software (Courage + Khazaka Electronic GmbH, Cologne, Germany). The principle of the measurement was based on the capacitance behavior of the skin appear as a dielectric medium [31]. The probe consisted of a ceramic tile with many closely-spaced gold electrodes, which functioned as a capacitor for measuring 49 cm^2^ skin areas for 1 s using a frequency of 0.9–1.2 MHz with an accuracy of ± 3%. Briefly, the Corneometer probe was placed onto the skin, and a readings were taken at 1 s intervals for 20 s each, and at least 10 determinations were calculated. The results were reported as proportional value to the dielectric constant of the skin with variations according to its level of moisture. Data were given in arbitrary units (A.U.) ranging from 0 (very dry) to 120 (very wet). The corneometry value increased as the moisture content increased. The probe was calibrated before use.

##### Measurement of Skin Viscoelasticity

The skin viscoelasticity was assessed using a Cutometer MPA 580 with a probe diameter of 2 mm as previously described by Rahmanian-Schwarz et al. [32]. The measurement method is based on the principle of suction and elongation. Before measurement, the probe was checked and calibrated using a small calibration cap. The skin firmness and elasticity were observed at baseline and after test formulations [33]. To begin the investigation, measurement mode 1 of the Cutometer MPA 580 was selected, and the probe was placed onto the skin area tested with a constant negative pressure of 450 millibars (approximately 6.53 pounds/square inch). The test skin area was then drawn into an aperture of the probe. After 2 s, the negative pressure was switched off for 2 s for the skin to return to its original shape. The linear parameters obtained from the resulting curve were used to calculate the relative parameters of elasticity and viscoelastic ratio by Cutometer software. An overall elasticity (Ua/Uf) was calculated by dividing the immediate extensibility that occurs in the extension curve at a level of recovery at the end of the relaxation curve (Ua) by the maximum deformation of the skin that occurs during the measurement (Uf). Net elasticity (Ur/Ue) is calculated by dividing the elastic deformation recovery at the force end (Ur) by the early deformation of the skin (Ue). The skin firmness (Ur/Uf) was calculated by dividing Ur by Uf, respectively [33].

#### 2.8.3. In Vivo Toxicity Studies

##### Vaginal Smear Check

The vaginal smear check was performed as an indicator of systemic toxicity of the formulation by using the method reported by Malaivijitnond et al. [34]. The appearances of cornified cells from vaginal smears of the rats were used as an indicator of systemic estrogenic effects. Vaginal smears were collected daily from the treated rats from 8.00 a.m. to 9.00 a.m. The vaginal epithelium cells observed under a light microscope connected to an image analyzer (Nikon Eclipse E200, Nikon Instruments Inc., Melville, NY, USA) were classified into 3 types: leukocytes, nucleated cells, and cornified cells (Co). The representative cell types were determined by the majority cells from the same treated group and were expressed as a mean value as the following Equation (2): [35].

(2) $$\%\mathrm{Co}=\frac{\mathrm{Number}\;\mathrm{of}\;\mathrm{cornified}\;\mathrm{cells}}{\mathrm{Number}\;\mathrm{of}\;\mathrm{total}\;\mathrm{cells}\;(\mathrm{leukocytes}+\mathrm{nucleated}\;\mathrm{cells}+\mathrm{Cornified}\;\mathrm{cells})}\times 100$$

##### Uterus, Liver, and Kidney Weight Changes

The rats were sacrificed at the end of day 84 following the study treatment period, and their uteri, livers, and kidneys were carefully dissected. The uteri were stripped of any adhering fat and connective tissue and placed on a filter paper. The fluid inside the uteri tubes was drained and weighed. The data were expressed as each organ to rat body weight ratio compared with those in the negative and positive control groups.

##### Primary Skin Irritation Studies

According to previous experiments and the literature-based data, primary skin irritation was assessed in rabbits following the International Organization for Standardization (ISO) 10993-10 [36,37]. The test formulations were applied to the dorsal skin of the animals. Single-dose applications of the test formulations were visually observed and photographed by a digital camera (Fuji Model Finepix S5500, Tokyo, Japan). The reaction that appeared on the skin was scored based on a grading scale from 0–4 according to the interpretation key of the International Contact Dermatitis Research Group [37,38]. The degree of skin allergy was given a numerical value of 0–4, where zero value indicates no reaction, ±1/2 a doubtful reaction, 1 a weak reaction, 2 a moderate reaction, 3 a strong reaction, and 4 a severe reaction.

### 2.9. Statistical Analysis

The data were expressed using mean ± standard deviation (SD). Statistically significant differences were determined using the Wilcoxon signed-rank test due to the small sample size. The H-test by Kruskal–Wallis was used for comparison between groups. In all cases, a minimal level of significance was set at *p* < 0.05 with the SPSS program (version 12, SPSS Inc., Chicago, IL, USA).

## 3. Results

### 3.1. Amount of Isoflavone Aglycones in FSE

**Figure 1:**
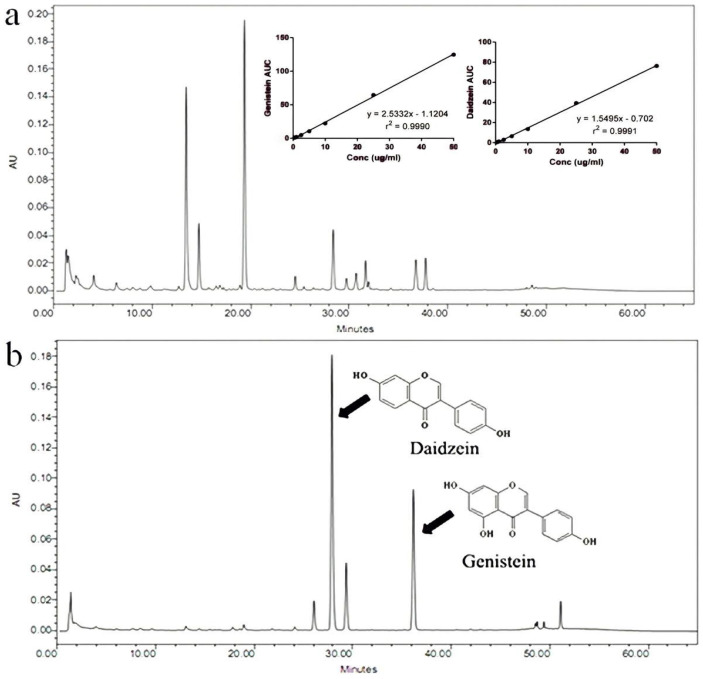
HPLC chromatograms of (**a**) non-fermented soybean extract and (**b**) *A*. *oryzae* TISTR 3018-fermented soybean extract (FSE).

Figure 1a,b shows HPLC chromatograms of non-FSE and FSE, respectively. Daidzein was first eluted at retention time (tR) of 30.32 min followed by genistein at 36.78 min. The isoflavone glycosides (tR of daidzin = 13.84 min, tR of genistin = 19.67 min) were found to be the major components in non-FSE (Figure 1a). On the contrary, fermentation of soybean with *A. oryzae* for 10 days showed near completion in the conversion of isoflavone glycosides into their corresponding aglycones (Figure 1b). Amounts of daidzein and genistein in FSE were 36.00 ± 2.24 and 16.52 ± 1.10 mg/g dried extract, respectively.

### 3.2. Estrogen-Like Activities of FSE

**Figure 2:**
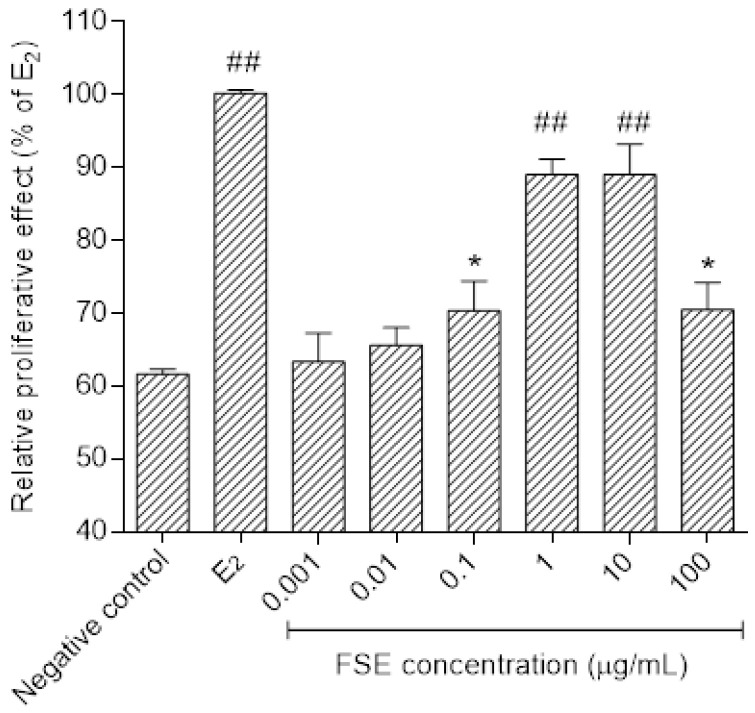
Estrogen-like activities determined in MCF-7 cells of FSE, * *p* < 0.05, ^##^ *p* < 0.001 compared to negative control (C). Each value represents the mean ± SD, *n* = 3.

**Figure 3:**
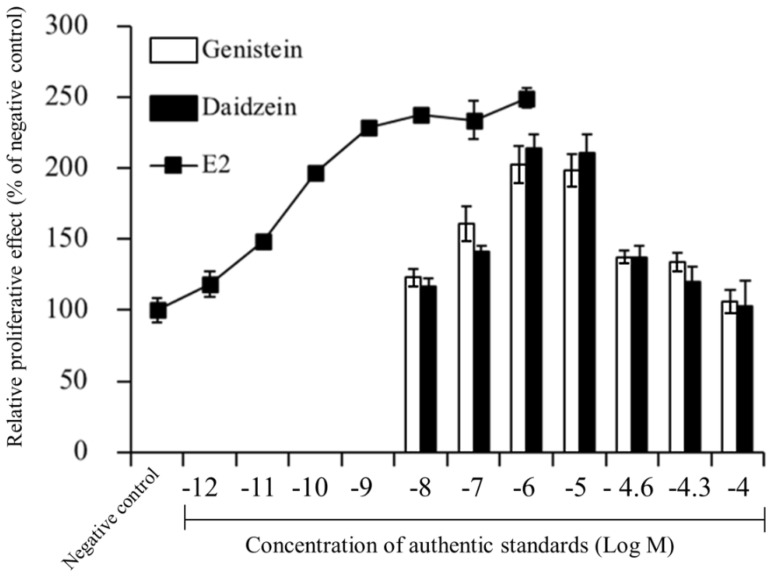
Estrogen-like activities determined in MCF-7 cells of E_2_, genistein and daidzein, *p* < 0.01, *p* < 0.001 compared to negative control (C). Each value represents the mean ± SD, *n* = 3.

As shown in Figure 2, FSE was able to significantly stimulate the MCF-7 cell proliferation in a dose-dependent manner (*p* < 0.05). The stimulatory effect on growth of the cells could be observed at concentrations of FSE starting from 0.001 to 100 μg/mL. Maximally proliferative effect of E2 is observed. At concentration of FSE 100 μg/mL, FSE suppressed cell growth. Effects of daidzein and genistein on the growth-promoting activity were also evaluated in the same cells. As expected, these isoflavones were able to stimulate the growth of MCF-7 cells at low concentrations, whereas the inhibitory effect of the cells was observed at high concentrations (Figure 3). Daidzein stimulated the growth of MCF-7 cells starting from 10^−7^ M up to 10^−5^ M and was comparable to genistein (*p* > 0.05). These isoflavones exhibited highest in growth stimulatory effect at 10^−6^ M while at higher concentrations than 10^−5^ M, it suppressed cell growth. However, the authentic isoflavones showed a lower potency in producing estrogenic effects compared to E2.

### 3.3. Characterization of Niosomes

The physicochemical characteristics of niosome formulations was shown in Table 1. he average particle sizes of FSE microniosomes and FSE nanoniosomes measured by PCS were 1035 ± 43 nm and 285 ± 13 nm, respectively. The zeta potential values of the niosomes were from −28 to −53 mV. For E2 nanoniosomes, the particle sizes of this formulation were 283 ± 8 nm with zeta potential values of −31.14 ± 3.11 mV. The surface charges of niosomes were expected to depend on some components of FSE such as proteins and fatty acids.

The developed FSE niosome formulations could entrap very high contents of daidzein and genistein of above 90%. The %*EE* of daidzein and genistein in FSE microniosomes were 94.38 ± 2.22% and 99.11 ± 0.12%, which were comparable to those in nanoniosomes (daidzein = 92.47 ± 3.18% and genistein = 97.53 ± 2.10%). E2 nanoniosomes had the %*EE* of 99.01 ± 0.21% [32].

### 3.4. Effects of FSE on Skin Hydration

**Figure 4:**
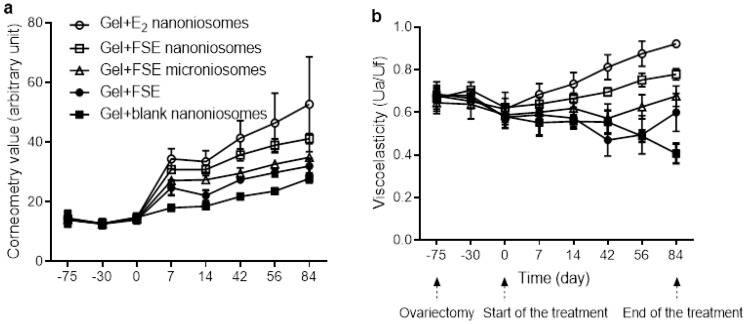
Effects of dermal application of various FSE formulations on (**a**) skin hydration and (**b**) viscoelasticity in the OVX rats. Values are expressed as mean ± SD (*n* = 4–5).

The effects of FSE on skin hydration measured before and following dermal application of the test gels are shown in Figure 4a. The effects of blank nanoniosomes and E2 nanoniosomes based gels are also given for comparison. No difference in baseline values was observed for all groups at pre-treatment period (*p* > 0.05). As expected, the skin hydration increased following FSE treatment for the test groups throughout the study period. These changes could be ranked from the highest to the lowest order as follows: gel + FSE nanoniosomes, gel + FSE microniosomes, gel + FSE. At the end of treatment (84 days), application of gel + FSE nanoniosomes showed a 1.48-fold increase in skin hydration, while gel + FSE microniosomes and gel + FSE were lower potencies with 1.26-fold and 1.15-fold increases, respectively, when compared to the gel + blank nanoniosomes. The change in corneometry values after topical application of the gel + FSE nanoniosomes was similar to the E2 group. The skin hydration of the OVX rats that received gel + blank nanoniosomes showed a slight change in corneometry values.

### 3.5. Effects of FSE on Skin Viscoelasticity

Figure 4b showed the plots between the Ua/Uf values against times of the study period. During the pre-treatment period, the baseline of Ua/Uf value for all groups showed similar values with no significant differences between groups at the same time point (*p* > 0.05). The Ua/Uf parameter in gel + blank nanoniosomes decreased compared to their baseline values up to the end of treatment day 84. In the rats that received gel + FSE, the Ua/Uf values slightly increased when compared to a negative control. This parameter increased significantly on day 84 of treatment (*p* < 0.05). In contrast, the Ua/Uf values in the rats received gel + FSE microniosomes and gel + FSE nanoniosomes significantly increased after day 56 and 14 of treatment, respectively. Additionally, it was obvious that the Ua/Uf values were more rapidly reversible in the E2 treated group. 

### 3.6. Vaginal Cornification Assay

**Figure 5:**
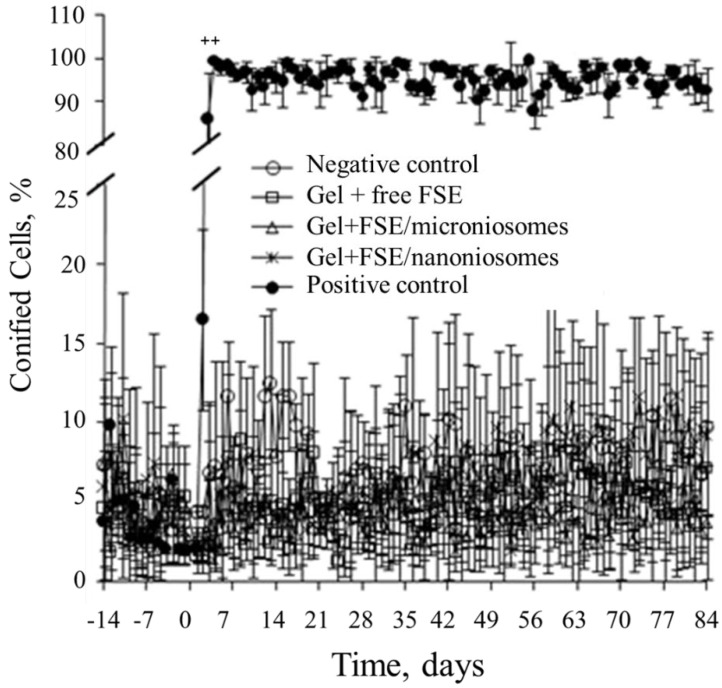
Percentage of vaginal cornified cells in rats treated with FSE for 84 days. *p* < 0.001 compared to negative control. Values are expressed as mean ± SD (*n* = 4–5).

Changes in vaginal cornification of each rat were checked daily to establish a baseline for 14 days prior to treatment and monitored cellular differentiation during treatment period (days 1–84). In the negative control group, vaginal cornification fluctuated in the range of 1.76–11.62% throughout the study period (Figure 5). Similar to the negative control group, there was no significant change in the level of vaginal cornification in all groups treated with various FSE-based gels when compared to their baselines with the percent of cornification being lower than 11.63% (*p* > 0.05). On the contrary, the percentage of vaginal cornification significantly increased in the estrogen group after the first 3 days of treatment with the percentage of cornified cells between 85.9–99.6% (*p* < 0.001). The population of vaginal cells in this group exhibited a distinct pattern beginning from leukocytes to nucleated cells and terminating as fully cornified cells.

### 3.7. Effects of FSE on Weights of Uteri, Livers and Kidneys

Changes in the relative weight of the uterus, liver and kidney of rats were further examined at the end of treatment to confirm toxicity of various FSE-based gels. As shown in Table 2, no significant differences in the relative weight of these organs could be observed between groups receiving FSE-based gels (*p* > 0.05). These relative weights were also comparable to that of the negative control group, indicating they were safe products. A contrary result was observed when the OVX rats received gel + E2 nanoniosomes. This potent hormone dramatically increased the uterus, liver and kidney weight of the rats, by almost 4.16-, 1.44-, and 1.27-fold as compared with the negative control group (*p* < 0.01), respectively.

### 3.8. Primary Skin Irritation Tests

The primary skin irritation indices obtained after formulation applications was examined on healthy white male New Zealand rabbits. The male rabbits were selected for this study because they have less fluctuation in estrogen hormones than the females. The negative control group received 0.9% *w*/*v* sodium chloride solution (20 μL/chamber) and showed no signs of skin erythema or edema (Table 3). The score of primary irritation (SPI) and skin irritation index (SII) was 0 and 0, respectively (negligible irritation). Similar results were also found for animals that received gel base, gel containing blank nanoniosomes, gel containing FSE, gel containing FSE/microniosomes, gel containing FSE/nanoniosomes or gel containing E2/nanoniosomes. In contrast, the animals receiving 5% *w*/*v* sodium lauryl sulfate solution exhibited erythema at the test sites and their SPI and SII were both 0.5, indicating mild irritation. The erythema of skin in the positively control animals remained stable for more than 72 h. These preliminary results suggest that the developed FSE products did not cause primary skin irritation and should be considered as safe.

## 4. Discussion

The isoflavone glycosides in soybean flour were converted into their corresponding aglycones by fermentation with *A. oryzae*. This may be from the catalytic activity of β-glucosidase secreted by the filamentous fungus during the fermentation as described elsewhere [12,13]. The obtained FSE containing predominant isoflavone aglycones was chosen as the ingredient for an anti-skin-ageing product. The E-screen assay showed that the FSE, daidzein, and genistein exhibited weak estrogenic activity compared to E2. Genistein and daidzein exhibited estrogenic properties in MCF-7 cells between 0.01–10 µM, while the anti-estrogenic effect was observed at higher concentrations (>10 µM). The results of this study were in accord with those reported by Maggiolini et al. [39] that high concentrations of genistein induced down-regulation of both ERα mRNA and protein level in MCF-7 cells. Kuiper at al. [40] suggested that the estrogenic activity of phytoestrogens depended on their affinity for ERs. This modulates the transcription of target genes in a variety of organs. They found that the relative binding affinities of genistein and daidzein to E2 for ERβ were 0.7 and 0.2%. On the contrary, affinities for ER-α were reported at approximately 13 and 1% for genistein and daidzein, respectively.

**Figure 6:**
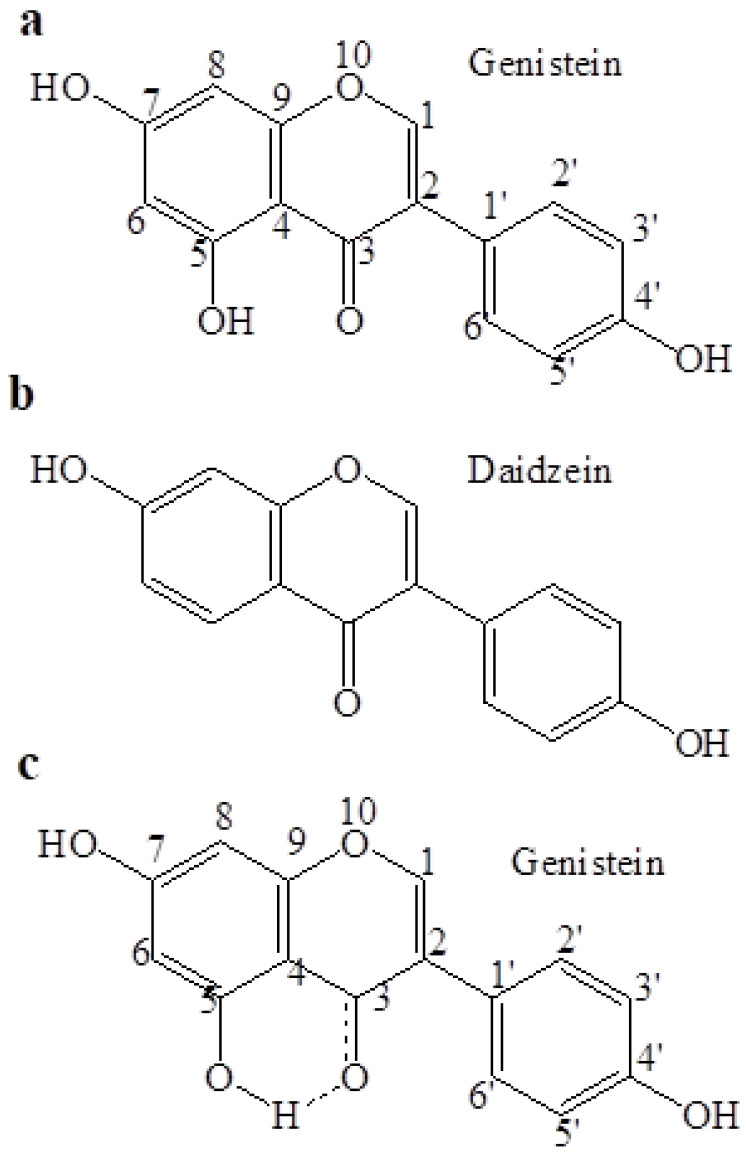
Chemical structures of (**a**) genistein, (**b**) daidzein, and (**c**) a possible chemical bond between the hydrogen atom at C_5_ and carbonyl group at C_3_ of genistein.

The chemical structures of genistein and daidzein are shown in Figure 6a,b, respectively. The %*EE* of genistein in both micro-and nanoniosomes was higher than that of daidzein. A possible explanation should be due to possible chemical bonds between hydrogen atoms at C5 of genistein with a carbonyl group at C3 (Figure 6c), leading to more lipophilicity than daidzein. Thus, a higher %*EE* of genistein could be attributed to its high interaction with the hydrophobic bilayer membrane of niosomes.

The ovariectomized rats were chosen as an alternative model to mimic skin ageing in post-menopausal women due to their short life time span and adequate time to obtain signs of pre-mature aging following the ovary removal. This is confirmed by results described by other groups that the OVX rats developed skin aging by the decrease in collagen content and mechanical properties of the skin [41,42]. The test gels containing FSE were shown to be efficacious in the improvement of skin hydration and viscoelasticity. Estrogen-like effects of the isoflavones could be, at least in part, the substances for improving biophysical skin properties via estrogenic receptor pathway. This is because isoflavone aglycones analyzed by HPLC were the major components of FSE. The previous study has been reported that an ERβ was a predominant form found in the skin, and it was strongly detected in the epidermis, eccrine glands, sebaceous glands, and hair follicles, while an ERβ was lower density [43]. However, the efficacy for improving the skin properties of FSE were lower than that of E2. This could be attributed to different estrogenic potency for both ER subtypes that lower activity for isoflavones. 

A combination of isoflavone mixtures might exhibit more efficacies than single compound due to a eutectic behavior of isoflavone extract leading to improved mobility, solubility, and permeation of such phytoestrogens through the skin [44,45]. Capability for improving the biophysical skin properties of FSE was in a particle size-dependent manner. The skin enhancement properties of FSE loaded into nanoniosomes exhibited greater efficacies than those in microniosomes or those in a conventional gel. These results would be explained by the effects of small sizes of niosomes, which facilitated skin penetration [46]. Encapsulation of FSE into nanoniosomes composed of non-ionic surfactants as the main components increased the concentration of isoflavones at the viable layers of the skin higher than other formulations. These layers of the skin are also well known as the site of action of phytoestrogens. The improvement in biophysical skin properties could result from higher concentrations of isoflavones derived from such an extract penetrating the skin and exerting their biological effects on the target cells.

Changes in corneometry generally reflect hydration of the skin. Positive effects were observed following treatment of the OVX rats with FSE in all groups. Interestingly, gel + FSE nanoniosomes showed the highest efficacy for increasing skin hydration. This could be explained by the influence of nanoniosomes for the delivery of the isoflavones into the target skin layers. Moreover, the obtained results also supported our in vitro previous findings that FSE nanoniosomes could improve the accumulation of phytoestrogenic makers (daidzein and genistein) into the deeper skin layers [47]. Trans-follicular route should act as an essential way involved in isoflavone absorption since the rats have high hair density. An increase in corneometry values may be caused by the isoflavones’ enhanced synthesis of hyaluronic acid in the skin. One study in mice reported that the glucosaminoglycan content markedly increased within two weeks of estrogen therapy [48]. This should be the reason why both FSE and E2 could improve skin moisture in the short time period after formulation application. This is confirmed by the results reported by previous studies [49,50] that gel-containing Bifidobacterium-fermented soy milk extract or pure isoflavone (genistein or daidzein) enhanced the production of hyaluronic acid in the skin, resulting in improvement of skin hydration and viscoelasticity in rodent model. This result is in good agreement with findings previously reported by Miyazaki et al. [50], who found that topical application of BE restored changes in the elasticity of mouse skin and human skin.

Post-menopausal women’s skin becomes dryer, thinner, wrinkling, and decreased in elasticity [3] and the ovariectomized rats have been used as a model to study estrogenic hormone replacement effects, of many compounds [36,51,52,53,54]. Decreasing the collagen content is one of the significant factors contributing to the loss of skin viscoelasticity. The skin viscoelasticity slightly decreased following extension times after ovariectomy, as seen in the pre-treatment period. The sign of skin aging became noticeable as the skin viscoelasticity of the rats treated with gel + blank nanoniosomes reduced by approximately 40% over the 75 and 84 days following the ovariectomy (Figure 4b). This skin aging implies that the declining level of estrogen in the post-menopausal period led to changes in the structural architecture of the skin and reduced skin biophysical parameters, including skin viscoelasticity. In the OVX-rats model, ovarian hormones are removed abruptly, while in natural menopause, there is a gradual alteration of hormones that start to occur at the perimenopause period and changes seen in the transition phase before reaching post-menopause [55]. Thus, the changes in rate and extent of skin viscoelasticity in OVX rats may not be comparable to humans. However, similar results reported a sharp decline in skin thickness and collagen in the years following menopause, particularly in the initial postmenopausal years [56].

However, these mechanical properties were found to be reversed back after topical application of FSE or E2 for the 84-day treatment period. There was specific evidence that demonstrated skin collagen could be improved or synthesized after topical estrogen or soy isoflavone therapies [3,6,7,57]. Incorporating the E2 into nanoniosomes had a more pronounced effect on the improvement of skin properties than that of FSE. Similar results have been reported in other [58], reporting that 1 week following topical application of non-fermented soybean extracts, approximately 5.7 μg/cm^2^ stimulated procollagen I and III expressions in the aged skin of post-menopausal women. Polito et al. revealed that dietary genistein aglycone significantly increased transforming growth factor-beta (TGF-β) and vascular endothelial growth factor (VEGF) as a result of increasing skin collagen of ovariectomized rats [54]. This result suggests that the improvement in the skin viscoelasticity could be attributed to phytoestrogens in FSE or E2 exerted their activities through the same molecular pathway occurring in other non-reproductive tissues. 

The vaginal cornification assay has been well-accepted as a sensitive method to predict the estrogenic activity for both phytoestrogens and estrogens [32]. This assay was selected as a biological indicator to monitor the systemic effects of tested gels. It is a non-invasive method that can be checked daily in each rat. There were no changes in vaginal cornification of the treatment groups compared to the negative control. This result should be due to either poor absorption of isoflavones from the skin to blood circulation or low potency of those substances in the mixture of FSE. A previous study conducted using Trifolium pretense as a source of phytoestrogens demonstrated that a low dose of the plant powder administrated orally did not induce the cornification of the rat’s vaginal cells [59]. However, an increase in the relative weights of the uteri, livers, and kidneys, as observed in the E2 group, could be caused by increasing the E2 content in the blood circulation. These findings suggest that no significant adverse effects of the test gels were observed, while higher toxicity was found for E2. Thus, FSE nanoniosome-based gel should be considered as a safer product with no systemic repercussions. These results agreed with recent data reporting that topical application of a conventional gel containing isoflavones [6] or oral administration of soybean extract [3,6] in postmenopausal women remained unchanged in their hormonally vaginal cytology. Additionally, the effects of positive control (E2) were in good agreement with results reported by Kaari et al. [60], but contradicted the results of Drews-jr et al. [61]. This was because the E2 nanoniosome-based gel increased in superficial and intermediate vaginal cells. The possible explanation could be attributed to such a potent hormone leading to blood circulation and exerting its biological effect on the vaginal epithelial cells and the uteri of the OVX rats. Moreover, as discussed above, soy-isoflavone exerts a higher binding affinity to Erβ. While many reproductive organs, including mammalian gland, ovarian, endometrium, and uterine epithelium, stimulate cell proliferation through ERα expression [62,63], ERβ shows that they counteract ERα stimulation of cell proliferation [64].

Using FSE in topical products has several advantages, including ease of use or the ability to avoid the first-pass metabolism of the isoflavones. However, the initial irritating inflammatory response should be kept in mind. Thus, the primary skin irritation of the test gels was also evaluated. Male New Zealand white rabbits are a good model for assessing primary skin irritation because they are susceptible to irritating agents and are widely used in pharmaceutical and cosmetic fields. In the positive control group, the test area of rabbit skin treated with sodium lauryl sulfate showed higher reactivity with signs of skin redness when compared to the negative control area. Concerning various FSE based gels, the rabbits were well tolerated without any evidence of skin irritation. This was probably related to the low skin irritation potential of the test gels.

However, it should be kept in mind that the obtained results were conducted in the OVX rat model, which tends to be more permeable to chemicals than human skin. Thus, randomized, blinded, and controlled clinical trials should be further studied in postmenopausal women to substantiate its dermal application. Additionally, using phytoestrogens should be avoided in patients with breast and endometrial cancer due to insufficient information on their safety.

## 5. Conclusions

The isoflavone glycosides were conversed to daidzein and genistein by fermentation of the soybean seeds with *A. oryzae TISTR3018*. The obtained FSE with moderate estrogen-like activity was chosen as the active ingredient for anti-skin-aging. Particle sizes of niosomes were the crucial factors that influenced biophysical skin properties. The FSE nanoniosome gel improved more efficacious skin hydration and viscoelasticity of the OVX rat than others, but displayed lower potency than E2. However, the former formulation showed good performance as it caused no vaginal cornification and changes in the weights of uteri, kidneys, and livers. The benefits of FSE to improve signs of skin aging should, at least in part, involve a potential phytoestrogenic activity. These findings suggest that topically applied FSE into micro-and nanoniosomes has potential anti-skin-aging effects caused by estrogen deficiency, with no systemic side effects.

## Figures and Tables

**Table 1:** Physicochemical characteristics of niosome formulations.

Formulation	Size (nm)	PDI	Zeta Potential	*EE* (%)	
			(mV)	Daidzein	Genistein
Blank microniosomes	1.047 ± 28	0.80 ± 0.21	−28.77 ± 2.17	−	−
1%FSE/microniosomes	1.035 ± 43	0.78 ± 0.14	−47.43 ± 9.61	94.38 ± 2.22	99.11 ± 0.12
Blank nanoniosomes	288 ± 16	0.24 ± 0.15	−30.11 ± 4.35	−	−
1%FSE/nanoniosomes	285 ± 13	0.22 ± 0.21	−53.4 ± 13.28	92.47 ± 3.18	97.53 ± 2.10
0.01%E_2_/nanoniosomes	283 ± 8	0.21 ± 0.18	−31.14 ± 3.11	E_2_ 99.01 ± 0.21	

Data are expressed as the means ± S.D., *n* = 3.

**Table 2:** Relative uterus, liver and kidney weights of rats treated by FSE microniosomes and nanoniosomes skin application after 84 days.

Formulation	Relative Organ Weight (mg/100 g Body Weight)		
	Uterus	Liver	Kidney
Gel + 1% free FSE ^a^	54.61 ± 3.91	3148 ± 570	549.42 ± 68.54
Gel + 1% FSE/microniosomes ^a^	50.23 ± 5.29	3509 ± 372	545.97 ± 12.21
Gel + 1% FSE/nanoniosomes ^a^	55.97 ± 0.86	3439 ± 527	565.72 ± 39.19
Negative control ^b^	52.31 ± 2.46	3240 ± 417	559.10 ± 34.59
Positive control ^b^	217.40 ± 7.90 ^‡^	4676 ± 269 ^‡^	708.03 ± 12.34 ^#^

All data are presented as the mean ± S.D., ^a^ *n* = 5, ^b^ *n* = 4. Data with ^#^ indicates significant differences from the negative control group at *p* < 0.01 and data with ^‡^ indicates significant differences from the negative control group at *p* < 0.001.

**Table 3:** Skin irritation scores after patch test of FSE microniosomes and nanoniosomes in rabbits.

Testing Group	Erythema			Edema			Score	
	24 h ^a^	48 h ^a^	72 h ^a^	24 h ^a^	48 h ^a^	72 h ^a^	SPI	SII
Base gel	0 ^d^-0 ^e^	0-0	0-0	0-0	0-0	0-0	0	0
Gel + blank nanoniosomes	0-0	0-0	0-0	0-0	0-0	0-0	0	0
Gel + 1% FSE	0-0	0-0	0-0	0-0	0-0	0-0	0	0
Gel + 1% FSE/microniosomes	0-0	0-0	0-0	0-0	0-0	0-0	0	0
Gel + 1% FSE/nanoniosomes	0-0	0-0	0-0	0-0	0-0	0-0	0	0
Gel + 0.1% E2/nanoniosomes	0-0	0-0	0-0	0-0	0-0	0-0	0	0
0.9% *w/v* NaCl solution ^b^	0-0	0-0	0-0	0-0	0-0	0-0	0	0
5.0% *w/v* SDS solution ^c^	1-1	1-1	1-1	0-0	0-0	0-0	0.5	0.5

^a^ Time after patch removal from the skin; ^b^ Negative control; ^c^ Positive control; ^d^ left side; ^e^ right side.

## Data Availability

The data presented in the manuscript is available on request from the corresponding author.
